# *QUANTAS*: a Python software for the analysis of thermodynamics and elastic behavior of solids from *ab initio* quantum mechanical simulations and experimental data

**DOI:** 10.1107/S1600576722000085

**Published:** 2022-02-10

**Authors:** Gianfranco Ulian, Giovanni Valdrè

**Affiliations:** a Department of Biological, Geological and Environmental Sciences, University of Bologna, Piazza di Porta San Donato 1, Bologna, 40126, Italy

**Keywords:** *QUANTAS* computer program, solid phases, thermodynamics, equations of state, second-order elastic constants, quantum mineralogy

## Abstract

Mineralogy, petrology and materials science are fundamental disciplines not only for the basic knowledge and classification of solid phases but also for their technological applications, which are becoming increasingly demanding and challenging. Characterization and design of materials are of utmost importance and usually need knowledge of the thermodynamics and mechanical stability of solids. Alongside well known experimental approaches, in recent years the advances in both quantum mechanical methods and computational power have placed theoretical investigations as a complementary useful and powerful tool in this kind of study. In order to aid both theoreticians and experimentalists, an open-source Python-based software, *QUANTAS*, has been developed. *QUANTAS* provides a fast, flexible, easy-to-use and extensible platform for calculating the thermodynamics and elastic behavior of crystalline solid phases, starting from both experimental and *ab initio* data.

## Abstract 2

This paper presents *QUANTAS*, an open-source Python-based software aimed at providing a fast, flexible and easy-to-use framework to calculate the thermodynamics and elastic properties of crystalline solids. *QUANTAS* could be of use for researchers involved in various fields of solid-state chemistry, physics and mineralogy.

## 1. Introduction

An important issue for materials scientists (mineralogists, petrologists, solid-state chemists and physicists, and materials engineers) is to obtain good-quality data of the physical and chemical properties of a solid phase at different pressure and temperature conditions. This fundamental knowledge can drive further research on both the thermodynamic stability of the system under investigation, for example the construction of phase diagrams, and possible innovative specific technological applications.

Among several experimental methods, two approaches are commonly employed to study the elastic properties of a solid material: (i) hydrostatic compression and (ii) uniaxial/biaxial deformation. In the first case, the mechanical data on the solid can be obtained from the variation of unit-cell volume/axis lengths with pressure (*e.g.* by using diamond-anvil cells) by means of equation-of-state (EoS) fitting. Such experiments are conducted either at constant temperature or by varying it during diffraction experiments (Fukui *et al.*, 2003 ▸; Gatta *et al.*, 2013 ▸, 2015 ▸; Pawley *et al.*, 1995 ▸). The greatest challenge for this kind of investigation is to describe the structural dynamics on simultaneous variation of both pressure and temperature, which still requires very complex experimental setups. In the second case, the stress–strain relationship allows the determination of the fourth-rank elastic tensor of the material, whose components are also called ‘elastic moduli’ and ‘second-order elastic constants’ by physicists and engineers, respectively. The analysis of the elastic properties can reveal many details about both single-crystal and polycrystalline behaviors and is of utmost importance for designing materials with tailored mechanical properties. Several methodologies could be employed to obtain the elastic moduli, such as Brillouin spectroscopy (Jiang *et al.*, 2006 ▸) and shock wave experiments (Duffy *et al.*, 1991 ▸; Yoon & Katz, 1976 ▸).

In the past two decades, quantum mechanical (QM) methods have been increasingly applied in solid-state and materials science fields for both simple and complex systems. For instance, in mineralogy, this new scientific branch even started being referred to as ‘quantum mineralogy’. The use of quantum mechanical approaches is mainly for structural, electronic, vibrational and catalytical (surface) properties. The simulation of the thermomechanical behavior of solids is only recent, principally because of the high computational costs required by such calculations. The basis to obtain thermomechanical insights of crystals is given by the quasi-harmonic approximation (QHA), as described by Anderson (1995 ▸). The QHA offers a simple formulation valid for several solids presenting different bond types, such as pure covalent (Ottonello, Zuccolini & Civalleri, 2009 ▸), mixed covalent–ionic (Ottonello *et al.*, 2010 ▸; Ulian & Valdrè, 2015*a* ▸), pure ionic (Erba, 2014 ▸) and mixed covalent–dispersive (Ulian & Valdrè, 2015*b* ▸,*c* ▸). This method, which is derived from the harmonic treatment of the unit cell of solids, introduces an explicit dependence of phonon frequencies on volume, thus overcoming the well known limitations of the harmonic approximation (HA) as reported by Baroni *et al.* (2010 ▸).

Several formulations describing the volume dependence of vibrational modes have been proposed: (i) Grüneisen’s mode-γ parameters (Anderson, 1995 ▸; Ottonello, Civalleri *et al.*, 2009 ▸); (ii) a volumetric polynomial fit of the thermodynamic quantities calculated by the HA (Prencipe *et al.*, 2011 ▸); and (iii) a direct polynomial fit of the individual phonon frequencies with respect to volume (Erba, 2014 ▸; Erba *et al.*, 2015 ▸). In all of these approaches, it is required to calculate the phonon modes of the solid under investigation at different unit-cell volumes. The main difference between the thermodynamic and phonon interpolation schemes resides in the number of fitting procedures to be performed, which is usually lower for the direct polynomial fit of the individual phonon frequencies. Typically, QHA calculations are carried out considering only hydrostatic pressure variations on the unit cell. However, it is known that phonon frequencies depend also on the changes in the cell parameters expressed as strains, as also reported and discussed in recent literature (Murri *et al.*, 2018 ▸; Ulian & Valdrè, 2018 ▸; Destefanis *et al.*, 2019 ▸). However, in the present work we refer to the most common approach to QHA.

Nowadays, there are various quantum chemistry codes that can calculate the quantities necessary to perform QHA and elastic analyses of solids. Phonon modes and elastic tensors can be obtained directly from the outputs of the quantum mechanical software thanks to automated algorithms implemented in the package and/or indirectly. For example, the elastic moduli require knowledge of the stress–strain relationship based on total energy calculations, which are performed for a systematic series of deformations of the crystal structure. Within this approach, the elastic moduli are related to the total energy of the solid via a Taylor expansion in terms of the strain components truncated to the second-order, as described by Perger (2010 ▸). In the simplest approach, it is necessary to (i) determine the number and type of deformations, (ii) calculate the total energy of the crystalline solid for each deformed cell, and (iii) numerically derive the elastic tensor components from the energy versus deformation curves. Both the *CRYSTAL* (Dovesi *et al.*, 2018 ▸) and the *VASP* (Kresse & Furthmüller, 1996 ▸; Kresse & Hafner, 1993 ▸) codes can provide the elastic moduli directly, because they implement routines that automatically perform the steps cited above. If the quantum mechanical code does not provide these routines, it is necessary to perform these operations by hand or rely on external codes/scripts that both generate specific input files for the QM software and analyze the output results, as performed for example by the *ElaStic* scripts (Golesorkhtabar *et al.*, 2013 ▸). The same applies for the calculation of the phonon modes required by QHA analysis and of the equation of state of the solid under analysis.

In addition, there is an increasing need of codes that can post-process data from both theoretical simulations and experimental measurements, to obtain other information such as the thermodynamic and thermoelastic properties, or the directional and averaged mechanical behavior. The available tools in the scientific community are usually very specific to just one or a few of the cited analyses, and a comprehensive and inclusive platform is still missing.

Starting from all these considerations, and trying to satisfy the needs of both experimentalists and theoreticians, we developed *QUANTAS* (acronym of ‘quantitative analysis of solids’), a software platform that can aid solid-state and materials scientists of different fields in obtaining a multiplicity of properties of a crystalline phase.

At present, *QUANTAS* allows the user to calculate

(i) the thermodynamic and thermoelastic properties of a material at selected pressure and temperature conditions from *ab initio* quantum mechanical results;

(ii) the equation of state of crystalline phases from both theoretical and experimental data;

(iii) elastic properties derived from the second-order elastic moduli, independently of the means used to obtain them.

The present work is organized as follows: Section 2 lists and briefly explains some of the extant codes to better compare their functions with those of *QUANTAS*. Section 3 reports and discusses the non-functional requirements of *QUANTAS*. Sections 4–6 [Sec sec6] show all the functionalities and implementations, and Sections S1–S4 in the supporting information present, for the sake of completeness, some test cases used to validate *QUANTAS*. Finally, possible future developments of the presented package are explained and discussed. This paper is not intended as a user manual but provides just some of the details that can be found in the online documentation (see Section 3). The focus is on the basic science, general concepts and engineering aspects of *QUANTAS*.

## 2. Extant software

In the following, we provide a brief presentation of other related codes, explaining the differences with the *QUANTAS* software.

### 2.1. Phonopy

*Phonopy* (Togo & Tanaka, 2015 ▸) is an open-source code written in Python (with some functions in C) which aims to calculate phonon properties of crystalline solids. It has several pre-processing routines, *e.g.* creation of input files (unit-cell structures) for the calculation of unit-cell energy related to atomic displacements, and post-processing ones, such as determination and plotting of phonon bands in *k* paths, specifically developed for first-principles simulations. *Phonopy* has several interfaces for different *ab initio* codes and is widely employed by quantum chemical researchers. It allows also the calculation of harmonic thermodynamic properties and includes a quasi-harmonic approximation framework. It employs a thermodynamic interpolation scheme, using either the Murnaghan (1937 ▸) or the Vinet (Vinet *et al.*, 1987 ▸) equation of state to minimize the volume at 0 GPa. In *QUANTAS*, we provide four equation-of-state formulations and polynomials to minimize the volume at different temperature and pressure states, and both thermodynamic and phonon interpolation schemes (see below).

### 2.2. EosFit7

The *EosFit* software (Angel *et al.*, 2014 ▸) is a copyleft suite of codes developed to calculate *P*–*V*–*T* EoSs and cell parameter variations with pressure *P* and temperature *T* from volume *V*, cell parameters and elasticity data (Milani *et al.*, 2017 ▸). The current version is based on the *CrysFML* Fortran library (Rodríguez-Carvajal & González-Platas, 2005 ▸, 2008 ▸) and includes both a console and a graphical user interface (Gonzalez-Platas *et al.*, 2016 ▸). It does not implement volume-integrated equation-of-state formulations that could be used by theoreticians to fit their total energy versus unit-cell volume curves.

### 2.3. ElAM

*ElAM* (Marmier *et al.*, 2010 ▸), implemented in Fortran 90, is an open-source command-line software that provides analysis of the second-order elastic tensor using well known solid-state physics formulations as described by Nye (1957 ▸). It has both 2D and 3D plotting capabilities (the first in PostScript format, the second in virtual-reality modeling language format). However, the software seems discontinued since the end of 2009. It does not provide the calculation of directional and averaged seismic wave speeds.

### 2.4. ELATE

A successor of *ElAM*, *ELATE* is an open-source online tool, entirely written in Python by Gaillac *et al.* (2016 ▸), which provides a detailed analysis of the second-order elastic tensor, together with both bi- and tri-dimensional plots. It is also integrated in the Materials Project, a large database of several properties of many solid phases. As for *ElAM*, no routine has been implemented to calculate the seismic wave speeds.

## 3. Non-functional requirements of *QUANTAS*

Many scientific software codes have very similar non-functional requirements, such as license type and programming language. For the development of *QUANTAS* we followed whenever possible the best practices for scientific code development that have been recently proposed by Wilson *et al.* (2014 ▸).

### 3.1. Software license

*QUANTAS* is released as a free and open-source code under the New Berkeley Software Distribution (BSD) software license. This aims to provide the necessary conditions for the verification and validation of research data (Joppa *et al.*, 2013 ▸), while also allowing advanced users to modify the source code, collaborate on future implementations, and ensure long-term maintenance and sustainability of the software.

### 3.2. Programming language

*QUANTAS* is developed in Python 3 (http://www.python.org), with the numerical sections implemented through the *NumPy* and *SciPy* scientific computing libraries (van der Walt *et al.*, 2011 ▸). Heavy computations are performed by a small portion of the software that was written in Cython (Behnel *et al.*, 2011 ▸), namely as C extensions for Python. The computation is in double precision for the thermodynamic/thermoelastic properties using the HA or QHA framework, whereas single precision is employed for the equation-of-state fitting and the analysis of the elastic tensor. The choice of a high-level language is motivated by its easier comprehensibility and maintenance, and also the need to provide cross-platform support. *QUANTAS* was developed keeping in mind the different environments where it could be employed, from desktop computers to servers. At the moment, the package is shipped as a Python library that can be installed by the user, but, in the near future, it will be available also from well known package repositories such as PyPI (https://pypi.org/).

### 3.3. User interface

*QUANTAS* uses a command-line interface (CLI), which is invoked by using a Python entry point. In general, a calculation is started from the console shell (or command prompt on Windows) by typing

quantas sub_command input_f ile_name [options]

The available sub-commands are related to the different routines implemented, whose names were chosen to be intuitive for users. For example, to run a quasi-harmonic approximation calculation, the sub-command that has to be called is qha.

In addition, a specific sub-command (inpgen) can be employed to generate input files for post-processing analyses of the (quasi-)harmonic approximation and second-order elastic constants:

quantas inpgen [generator] input_f ile [options]

This routine is particularly useful given the complexity of extracting the phonon modes from quantum mechanical simulations and formatting them for a *QUANTAS* input file.

### 3.4. Online services

The *QUANTAS* source code is hosted at https://github.com/gfulian/quantas, and detailed documentation on how to obtain (download), install and use the software is provided by the ReadTheDocs service (hosted on https://quantas.readthedocs.io). The latter is also home of some tutorials designed to guide users in creating the input files, running the different analyses and collecting the results. The web site and documentation were created using the *Sphinx* code (https://www.sphinx-doc.org/).

## 4. (Quasi-)Harmonic approximation

### 4.1. Theory

In quantum mechanical simulations of periodic three-dimensional systems (crystals), the harmonic thermodynamics of any system can be obtained from its phonon properties, which can be calculated just in the central point of the first Brillouin zone (Γ point) or in several **k** points in the reciprocal space. Several reports in the literature describe different approaches to calculating the Γ point (**k** = 0) vibrational modes and the phonon dispersion relations, whose detailed description is beyond the scope of the present work. The interested reader may refer to the fundamental work of Parlinski *et al.* (1997 ▸), Erba (2014 ▸) and Togo & Tanaka (2015 ▸).

Let us assume that, given a crystal unit cell, a certain number of **k** points have been sampled. It is known that 3*N* oscillators (phonons), with *N* the number of atoms in the cell, are associated with each considered **k** point. In turn, each phonon is associated with an energy level given by the harmonic expression 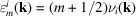 , with *m* an integer number and *ν_i_* (**k**) the frequency of the harmonic oscillator. It is then possible to express the vibrational canonical partition function of the system as

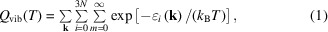

where *k*_B_ is the Boltzmann constant. Statistical thermodynamics defines the entropy *S*(*T*), the thermal internal energy *U*_th_(*T*) and the isochoric heat capacity *C*_*V*_(*T*) of a system as

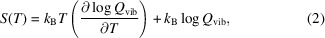




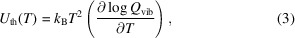




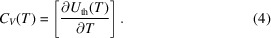

By substituting equation (1) into equations (2)–(4) [Disp-formula fd4], it is possible to write the harmonic expression of the thermodynamic properties as reported by Prencipe *et al.* (2011 ▸) as

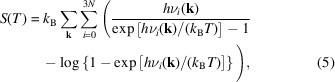




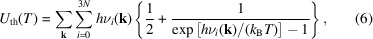




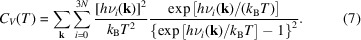

While the harmonic approach has been successfully adopted in predicting vibrational (spectroscopic) and thermodynamic properties of several systems (Belmonte, 2017 ▸; Erba, 2014 ▸; Prencipe *et al.*, 2004 ▸; Ulian *et al.*, 2021 ▸; Ulian & Valdrè, 2019 ▸), it suffers from several well known limitations. In fact, many important properties of crystalline materials are wrongly described within this framework; for instance, the elastic constants and the bulk modulus do not depend on temperature, the constant-pressure and constant-volume heat capacities are equal, and the thermal expansion is null. There are several methods that can add the contribution of volume (pressure) to the thermodynamics of a solid system, among which one of the most powerful is the QHA (Anderson, 1995 ▸). The QHA includes an explicit dependence of the vibrational phonons on the crystal volume, *i.e.* 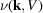 , in the harmonic description of the Helmholtz free energy:

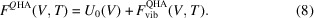

The Helmholtz free energy *F* ^QHA^(*V*, *T*) is the sum of the static energy of the system *U*_0_(*V*) at *T* = 0 K and the vibrational (thermal) contribution 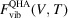 . *U*_0_(*V*) is obtained by any quantum mechanical code by geometry optimization of the unit cell at selected (and constrained) volumes. The thermal contribution 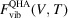 in the QHA term is defined as

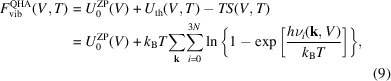

where 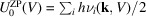 is the zero-point vibrational energy. From equation (9), it is possible to calculate the equilibrium volume at selected temperatures by minimizing the 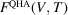 term with respect to volume. The volumetric thermal expansion coefficient α_*V*_(*T*) at selected pressure can be expressed as

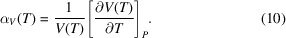

It is possible to describe the isothermal bulk modulus (*K_T_*) of the crystal from the energy second derivative of equation (8) at fixed temperature as

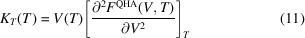

and also the adiabatic bulk modulus (*K_S_*), which is a preferred way to report the elastic behavior of the solid when comparing the theoretical results with experimental data obtained from some techniques (*e.g.* elastic wave analysis), as

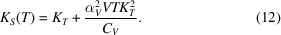

The great advantage of the QHA approach is the combination of pressure and temperature effects, as the pressure is calculated by

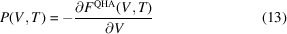

and knowing the temperature we can calculate all the other properties at selected *P*–*T* conditions.

From these formulations, it is possible to calculate the isobaric heat capacity of the system using the quantities obtained from equations (10) and (11) and from *V*(*T*):

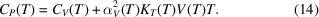




Finally, other thermodynamic properties could be calculated from the equations above, such as enthalpy and Gibbs free energy.

The quasi-harmonic treatment, as reported above, needs an adequate knowledge of the phonon dispersion relations, namely how the phonon modes vary with the **k** point in the first Brillouin zone. This is particularly relevant to properly describe the three acoustic phonon bands of any crystal system under consideration, because they represent the main contribution to thermodynamic properties at low temperatures (Belmonte, 2017 ▸), but they are always null at the Γ point (central zone, **k** = 0). For simple systems, *e.g.* containing from two to 20 atoms, phonon dispersion relations can be calculated with supercell approaches as described by Parlinski *et al.* (1997 ▸). However, such methods could become too computationally demanding for larger unit cells, and hence more approximate algorithms must be employed instead. From this perspective, it is possible to calculate the acoustic thermodynamic properties from sine wave dispersion relations as described by Kieffer (1979*a* ▸,*b* ▸,*c* ▸):

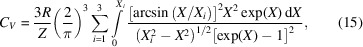




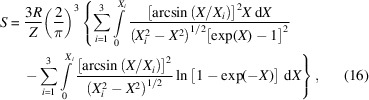

where *X* = *hν*/(*k*_B_ *T*), *R* is the gas constant, *Z* is the number of unit formulae per unit cell and the integrals are evaluated up to the acoustic phonon boundary, the latter deriving, for example, from second-order elastic moduli. Albeit approximate, this approach can provide the acoustic contributions to thermodynamic and thermoelastic properties with adequate accuracy for a system with more than 20–30 atoms in the unit cell (Prencipe *et al.*, 2011 ▸; Ulian & Valdrè, 2015*b* ▸; Ulian *et al.*, 2021 ▸).

### 4.2. Implementation

**Figure 1:**
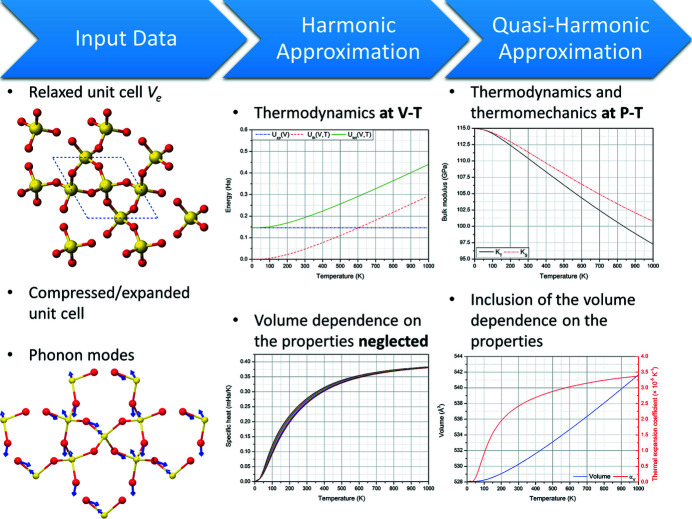
Overview of the *QUANTAS* approach for harmonic and quasi-harmonic approximations. The software employs quantum mechanical data from *ab initio* codes to calculate thermodynamic and thermomechanical properties of a solid phase at selected temperature and pressure conditions.

**Figure 2:**
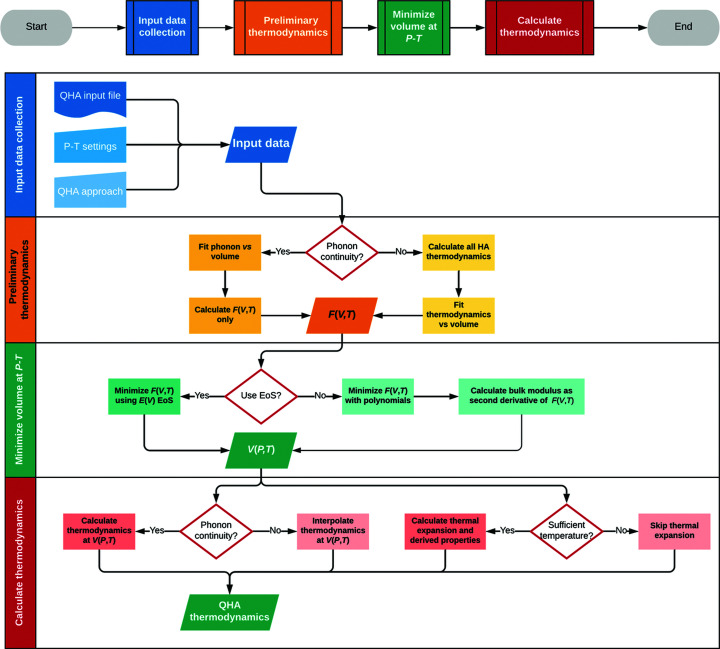
*QUANTAS* workflow of the quasi-harmonic approximation routine, with swimlanes providing specific details on how the input data are treated to calculate the quasi-harmonic thermodynamic results.

*QUANTAS* implements both HA and QHA methods for the analysis of thermodynamics, as summarized in Fig. 1 ▸. A scheme of the workflow specifically related to the quasi-harmonic approximation is provided in Fig. 2 ▸. Formulations based on both (i) the complete phonon dispersion relations and (ii) the approximated sine wave dispersion relations are included, which can be selected according to the available input data.

While the harmonic approximation routines are quite straightforward, *i.e.* they involve summations of quantities calculated over each phonon frequency for each unit-cell volume (see above), the QHA approach needs special care, as it deals with both fitting and minimization procedures (see Fig. 2 ▸). There are two critical points in the quasi-harmonic treatment:

(i) how the phonon/thermodynamics dependence on volume is described;

(ii) how the minimization of the Helmholtz free energy [ 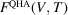 ] curves is performed.

#### 4.2.1. Phonon frequency dependence on volume

For the phonon frequency dependence on volume, two possible strategies are implemented: (i) exploiting an explicit dependence of the phonon modes on unit-cell volume and (ii) providing an implicit dependence using the harmonic approximation thermodynamics. In both cases, linear, quadratic or cubic polynomial functions can be employed. A functional description of the phonon versus unit-cell volume curves, *ν_i_* (**k**, *V*), should be the preferable option, as it allows the thermodynamics of the system to be calculated at each desired *P*–*T*–*V* condition from statistical thermodynamics.

However, to obtain the *ν_i_* (**k**, *V*) curves a correct description of the phonon frequency continuity over the explored volumes is required, taking into account possible crossing of the phonon bands by performing scalar products of the normal mode eigenvectors. In contrast, the employment of a functional (polynomial) form of the thermodynamic quantities over the volume (at constant *T*) is more straightforward than the former method (since vibrational frequency continuity is not necessary), but there are more fitting procedures involved (for each considered temperature, there are six polynomial fits). Our tests, and the results reported by Erba and co-workers (Erba, 2014 ▸; Erba *et al.*, 2015 ▸), suggest that the first method using the phonon continuity over volume provides results in very good agreement with experimental data, depending on the quantum mechanical approach employed to obtain the electronic energy and the vibrational frequencies. However, in *QUANTAS* both formulations were included, because some users may not be able to obtain (or use) the phonon mode eigenvectors to check their continuity over volume.

#### 4.2.2. Minimization of Helmholtz free energy

Two strategies were implemented in *QUANTAS* for the minimization of the Helmholtz free energy at each temperature value: (i) an equation-of-state fitting procedure of 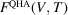 by means of volume-integrated formulations or (ii) minimizing an *n*th-order polynomial that fits 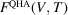 as a function of volume. This procedure leads to the equilibrium volume at selected pressure and temperature conditions. Generally, the first method is the preferred experimental way to obtain a phenomenological description of the system under hydrostatic compression, and its main advantage in the *QUANTAS* framework is that it yields with just one fit both the equilibrium volume *V*(*T*) and the bulk modulus *K_T_* (*T*) at a selected temperature. The equation-of-state formulations implemented in *QUANTAS* are the Murnaghan (1937 ▸), third-order Birch–Murnaghan (Birch, 1947 ▸), Vinet (Vinet *et al.*, 1987 ▸) and Poirier–Tarantola, also called ‘natural strain’ (Poirier & Tarantola, 1998 ▸), all of them in their volume-integrated form (Fu & Ho, 1983 ▸; Hebbache & Zemzemi, 2004 ▸). However, despite being more experimentally friendly, this approach may result in slight numerical noise at high temperature (higher than about 1500 K) in the thermomechanical data, whereas the adoption of a numerical (polynomial fitting) approach can provide more stable results. With polynomial fitting, the isothermal bulk modulus *K_T_* (*T*) can be computed according to equation (11).

The equilibrium volume at each desired pressure is obtained, either from EoSs or from polynomial functions, by minimizing

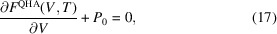

where *P*_0_ is the target pressure. The *V*(*P*, *T*) values are then used to calculate the thermodynamic and thermoelastic properties at any selected *P* *–T* conditions.

### 4.3. Input data for (Q)HA calculations

In order to use the routine proposed in the present work, some data from quantum mechanical simulations have to be obtained. *QUANTAS* is developed to be ‘code blind’, meaning that any QM code that uses its specific algorithms and measurement units could be used to produce the required input information.

The following input data from QM simulations are required:

(1) Starting bulk geometry (with volume *V*_e_) of the system under analysis, fully optimized for both lattice parameters and atomic coordinates.

(2) Several bulk structures relaxed in both compression and expansion regimes with respect to the equilibrium volume *V*_e_. A suitable approach is described in very recent literature (Erba *et al.*, 2015 ▸), which considers a number of volumes *N_V_* between *V*_e_ − *sV*_e_% and *V*_e_ + 2*V*_e_ *s*% (including *V*_e_), with *s* the step of the compression/expansion. Reliable values of *N* are 4, 7 and 13, and the step *s* can be set in the range 2–5.

(3) A complete set of vibrational frequencies (or phonon dispersion relations) for each geometrically relaxed unit-cell volume.

In the input file, the unit-cell volume and the lattice static energy (0 K, no thermal contributions) are provided as arrays of length *m* (the number of compressed/expanded volumes), and the phonon modes are reported as a matrix of shape *k* × *m* × *p*, where *k* is the number of sampled **k** points in the Brillouin space and *p* is the number of phonon modes (3*n*, with *n* the number of atoms in the unit cell). For example, the user could employ the Γ-point vibrational frequencies calculated for a unit cell containing 40 atoms, studied in five compression states: in this case, *k =* 1, *m =* 5 and *p* = 120, whereas *k* > 1 when phonon dispersion relations were simulated. Compared with the use of only Γ-point vibrational frequencies, the accuracy of the quasi-harmonic approximation results increases when phonon dispersion relations (*k* > 1) are employed as input data.

## 5. Equation of state

In both experimental and theoretical settings, the volumetric behavior of a solid phase with pressure can be described in a functional form called the ‘equation of state’. The volume of the solid is related to its unit cell, which could be obtained from high-pressure diffraction experiments. The equation of state is a parametrized function, containing from two to four parameters that are adjusted to fit the experimental data. There are several places in the literature where a detailed description of the theory behind the EoS formulations is provided (Anderson, 1995 ▸; Angel, 2000 ▸), and here only the relevant information is discussed.

At the moment, only five isothermal equation-of-state formulations are coded in *QUANTAS*:

(1) Murnaghan (1937 ▸):

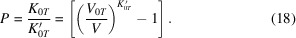




(2) Birch–Murnaghan (Birch, 1947 ▸):

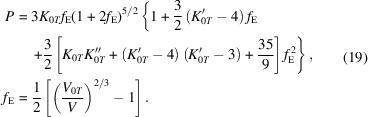




(3) Vinet (Vinet *et al.*, 1987 ▸):

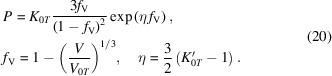




(4) Modified Tait (Freund & Ingalls, 1989 ▸):

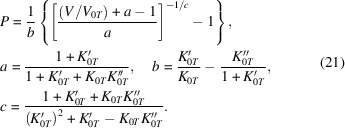




(5) Natural strain (Poirier & Tarantola, 1998 ▸):

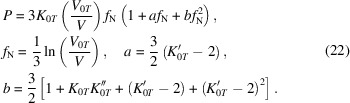

Here, *V*_0*T*_ is the unit-cell volume, *K*_0*T*_ is the bulk modulus, 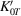 is the first derivative of the bulk modulus with respect to pressure and 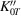 is the second derivative of *K*_0*T*_. The subscripts ‘0’ and ‘*T*’ mean that each parameter is obtained at reference pressure zero and temperature of interest *T*, respectively.

The modified Tait (T) and Murnaghan (M) EoSs are ‘invertible’ formulations, as it is possible to express the unit-cell volume as a function of pressure by inverting the equation. In addition, the modified Tait equation of state can be reduced to the Murnaghan one by imposing 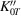 = 0.

The Birch–Murnaghan (BM) and natural strain (NS) equations of state are ‘finite-strain EoSs’, which were formulated considering that the energy of the compressed solid can be expressed as a Taylor series in the linear strain *f* (that is Eulerian strain, *f*_E_, for the BM EoS and natural strain, *f*_N_, for the NS EoS). Both of them are fourth-order expansions, but they can be truncated to third- and second-order expressions by using assumed values for the 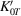 and 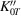 parameters, respectively, as described by Angel *et al.* (2014 ▸).

The Vinet (V) equation of state was derived from molecular mechanics models for very high compression regimes and is a third-order EoS.

According to the literature (Angel, 2000 ▸) and to the reported formulations, the fitting strategy considers the volume as the independent variable and the pressure as the dependent one, because the experimental uncertainties on *V* are generally much lower than those on *P*. Then, a least-squares method is used to fit the data, employing the errors on the variables as weights during the procedure. For example, this is the approach adopted by the well known *EosFit* software (Angel, 2000 ▸; Angel *et al.*, 2014 ▸). The goodness of fit is given by the residual variance (weighted chi squared, χ^2^), which is equal to unity if the EoS model perfectly matches the weighted experimental data. In contrast, if χ^2^ > 1 it means that the equation of state correctly represents only a portion of the data for several possible reasons, *e.g.* some compression states were not adequately obtained, the errors of the values were underestimated or the model is not accurate enough to describe all of the data set. For example, it is discouraged to use the Murnaghan EoS for unit-cell compressions higher than 10%. A value of χ^2^ < 1 does not represent a better fit and may also express an overfitting of the data, namely the equation-of-state model contains more parameters than the number of *P*–*V* points.

A visual assessment of the goodness of fit can also be obtained from the strain-normalized pressure plots (*f*–*F*), which are usually clearer than the standard *P*–*V* plots.

### 5.1. Implementation

While the physics behind the different formulations is well known within the community, the fitting procedure requires some explanation. Standard least-square procedures consider uncertainties only on the dependent variable, while the independent one is considered free from errors. In the EoS fitting, the dependent variable is pressure because the errors associated with pressure measurements are usually much larger than those of the unit-cell/lattice parameters. However, including the uncertainties on the unit-cell volume *V* would increase the accuracy of the fitted EoS parameters. Differently from *EosFit7* (Angel *et al.*, 2014 ▸), which employs the effective variance method described by Orear (1982 ▸), *QUANTAS* adopts the orthogonal distance regression (ODR) statistical approach (Boggs *et al.*, 1987 ▸), which allows the inclusion of the uncertainties of both dependent and independent variables. The ODR algorithm is coded in the *ORDPACK* library (Boggs *et al.*, 1989 ▸; Zwolak *et al.*, 2007 ▸), which is included in the *SciPy* Python package used by *QUANTAS*. If the user desires to weight the fit only for pressure, the software automatically switches to the ordinary least-squares procedure.

The fitting procedure is completely interactive, and users can have exact and complete control over it. As in *EosFit7* (Angel *et al.*, 2014 ▸), users may set weights and fix parameters and modify them to find the best EoS parameters for their data.

For this operation, the input file must contain the isothermal data of the material unit cell at different pressure states. These quantities can derive from either experiments or theoretical simulations. The input data are organized in a table-like format, indicating which quantity is present in each column. Uncertainties on both pressure and unit-cell volume/lattice parameters can be included.

## 6. Elastic properties from second-order elastic constants

In the theory of linear elasticity, the stress tensor can be expressed in terms of strain by

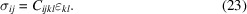


*C_ijkl_* are the components of the fourth-rank modulus tensor, whose coordinates depend on the choice of the axes. Equation (23) can be inverted, leading to

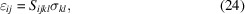

where *S_ijkl_* are the components of the compliance tensor, the inverse modulus tensor. A fourth-rank tensor has 81 components, but a maximum of 21 independent values, for triclinic crystals. It is also common to express the stiffness and compliance tensor components using the Voigt (engineering) matrix notation (see Nye, 1957 ▸), which is often adopted because of its simplicity. Note that the Voigt notation is not a tensor, but a matrix representation of it.

According to Nye (1957 ▸), several mechanical properties related to the polycrystalline (isotropic) elastic behavior of a material can be calculated from the elastic tensor by means of the Voigt, Reuss and Hill averages:

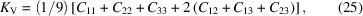




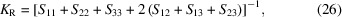




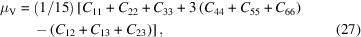




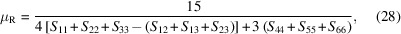




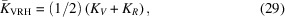




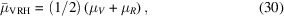




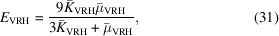

where *E*_VRH_ is Young’s modulus, and *K*_R_, *K*_V_, *K*_VRH_, μ_R_, μ_V_ and μ_VRH_ are the Voigt, Reuss and Hill values of the bulk and shear moduli, respectively.

The mean shear, 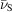 , and longitudinal, 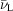 , wave speeds of a polycrystal with no preferred orientation of the grains depend on the coupling between grains and can range from the Reuss limit (with free grain boundaries) to the Voigt limit (with locked grain boundaries). Most randomly oriented polycrystals have shear and Young’s moduli close to, but not identical to, the VRH averages, for which the following approximation of the mean wave speeds is valid:

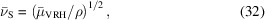




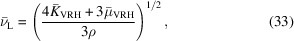

where ρ is the density of the crystal.

The calculation of the six eigenvalues of the second-order elastic tensor allows us to define the mechanical stability of the solid as described by Mouhat & Coudert (2014 ▸) for the different crystal systems: if any of the eigenvalues are negative, the system is unstable.

It is also possible to derive single-crystal elastic properties from the stiffness matrix, and an excellent treatment of their calculation was recently proposed by Marmier *et al.* (2010 ▸). In brief, this requires the transformation of the stiffness tensor using the following rule for generic fourth-rank tensors ***T***:

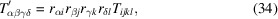

which employs the Einstein summation rule with the terms *r_αi_* being the direction cosines. In a Cartesian reference system, it is possible to represent a direction corresponding to an elastically significant distortion as a point on a unit sphere (unit vector **a**), using two angles, θ(0, π) and φ(0, 2π):

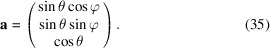

A single vector is sufficient to calculate the spatial variation of some properties, such as Young’s modulus or the linear compressibility, but a second vector **b**, perpendicular to **a**, is required to obtain the shear modulus and Poisson’s ratio. The second vector is characterized by a third angle, χ(0, 2π), and by the coordinates

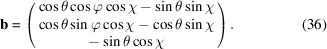

Then, the coordinates of **a** and **b** represent the first two columns of the rotation matrix, which allows the calculation of all the components in the subvectorial space defined by directions 1 and 2:

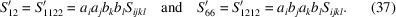

The spatial dependence of the elastic modulus *E* and linear compressibility β are defined as

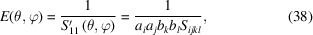




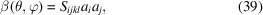

and the spatial variations of the shear modulus μ and Poisson’s ratio υ are given by the following formulae:

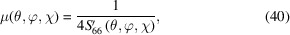




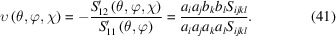

Directional variations of seismic (acoustic) wave speeds can be obtained by solving the Christoffel equation, as reported by Musgrave (1970 ▸).

### 6.1. Implementation

The analysis of the second-order elastic constant matrix is performed by a forked version of the *ELATE* script (Gaillac *et al.*, 2016 ▸), which was modified in our program to work offline and adapted to the *QUANTAS* framework. This modified version includes also a routine to find the reference crystal system and provides analysis of the seismic wave speeds by solving the Christoffel equations as reported by Musgrave (1970 ▸). Regarding the plotting capabilities, the original framework was changed to the *Matplotlib* library (Hunter, 2007 ▸), providing polar plots of the spatial variations of the elastic properties on the *xy*, *xz* and *yz* planes.

The elastic moduli can be provided to *QUANTAS* as an input file containing the stiffness matrix (*i.e.* the elastic tensor in Voigt’s notation) in either full, upper triangular or lower triangular form. The code performs a conversion from the matrix form to the tensorial form for the analysis of the elastic properties. This conversion preserves the Cartesian reference frame that was employed to obtain the elastic tensor in Voigt’s notation. If the crystal density is supplied, analysis of the seismic wave speeds is also performed. The analysis is automatic, and the user may ask to produce polar plots of the results.

## 7. Future development

For the future development steps of *QUANTAS*, we are working on new functionalities, for example, the capability to construct the phase diagrams for both a single substance (*e.g.* silica compounds) and solid solutions, by using the calculated Gibbs free energy values. This would resemble the CALPHAD method (Lukas *et al.*, 2007 ▸), but applied to the theoretical/experimental materials science fields. Concerning the EoS capabilities, it is planned to add thermal and *P*–*T* and *P*–*T*–*V* equations of state to aid the analysis of high-pressure and high-temperature data.

In addition, a graphical user interface is currently under development, with the aim of easing the use of the software and providing a plug-in platform that could be easily implemented with new functionalities.

Finally, since *QUANTAS* is an open-source project, we encourage users to contribute to our code, extending its functionalities with new modules.

## 8. Conclusions

There is a continuous and growing interest in the development and characterization of different materials at both experimental and theoretical levels, with important basic, technological and industrial applications. In this context, a detailed knowledge of the thermodynamic and elastic stability of solid phases is of utmost importance. In addition, thanks to increasing computational power, density functional theory is rising as a competing tool to drive innovation in materials science, physics, chemistry and other disciplines. Indeed, this framework offers an unprecedented balance between accuracy and speed.

The scope of *QUANTAS* is to provide a tool to reach a better understanding of both experimental and theoretical results, and to extend the knowledge on the *P*–*T* behavior of synthetic materials and minerals. *QUANTAS* is a fast easy-to-use software that can support researchers in speeding up their calculations, improving data quality over a wide range of cases. The software structure is simple and written in the Python programming language, which is truly cross platform.

As developers, we also encourage and support the integration of *QUANTAS* into other software that relies on quantum mechanical simulations of solid phases. The source code is flexible and reusable with little modification by other developers, a feature that is also facilitated by the distribution of the code under the Simplified BSD license.

Last, but not least, *QUANTAS* can be considered as a useful teaching software aid as well as a practical and powerful research tool. Students of various levels of study may find it helpful in understanding how thermodynamics and mechanical properties of materials behave and evolve.

## 9. Related literature

The following additional literature is cited in the supporting information: Anderson *et al.* (1991 ▸, 1992 ▸, 1993 ▸), Angel *et al.* (1997 ▸), Chopelas (1990 ▸), Comodi *et al.* (2002 ▸), Cynn *et al.* (1995 ▸), Gatta *et al.* (2006 ▸, 2014 ▸), Isaak *et al.* (1989 ▸), Kantor *et al.* (2012 ▸), Kresse & Joubert (1999 ▸), Lotti *et al.* (2017 ▸), Monkhorst & Pack (1976 ▸), Pascale *et al.* (2004 ▸), Peintinger *et al.* (2013 ▸), Perdew *et al.* (1996 ▸), Perger *et al.* (2009 ▸) and Tosoni *et al.* (2005 ▸).

## Supplementary Material

Explained and discussed test cases for the software. DOI: 10.1107/S1600576722000085/vb5027sup1.pdf

Archive containing the software and the related documentation (part 1). DOI: 10.1107/S1600576722000085/vb5027sup2.bin

Archive containing the software and the related documentation (part 2). DOI: 10.1107/S1600576722000085/vb5027sup3.bin

Archive containing the software and the related documentation (part 3). DOI: 10.1107/S1600576722000085/vb5027sup4.bin

Archive containing the software and the related documentation (part 4). DOI: 10.1107/S1600576722000085/vb5027sup5.bin

Archive containing the software and the related documentation (part 5). DOI: 10.1107/S1600576722000085/vb5027sup6.bin
