# Supplementary material for: QUANTAS: a Python software for the analysis of thermodynamics and elastic behavior of solids from ab initio quantum mechanical simulations and experimental data
Source: J Appl Crystallogr. 2022 Feb 10;55(Pt 2):386–96. doi: 10.1107/S1600576722000085 (PMC8985604; doi:10.1107/S1600576722000085)
Supplement: Supplementary file 1 [file j-55-00386-sup1.pdf]

# *QUANTAS*: a Python software for the analysis of thermodynamics and elastic behaviour of solids from *ab initio* quantum mechanical simulations and experimental data

**Gianfranco Ulian[a]* and Giovanni Valdrè[a]***

[a]Department of Biological, Geological and Environmental Sciences, University of Bologna, P. Porta San Donato 1, Bologna, 40126, Italy

Correspondence email: gianfranco.ulian2@unibo.it; giovanni.valdre@unibo.it

# Supporting information

In the following paragraphs, some examples of the results provided by each routine of *QUANTAS* are reported for a validation of the software.

### S1. Periclase MgO

Periclase is one of the main constituents of the mantle of the Earth and, because of its very simple structure (cubic), can be considered a prototypical mineral phase to assess the validity of computational models for the description of the high-pressure (HP) and high-temperature (HT) behaviour of minerals. Indeed, several works in literature reported the thermodynamic and thermoelastic properties of periclase (Isaak *et al.*, 1989, Chopelas, 1990), tests of thermodynamic theories employing this phase as a benchmark (Anderson *et al.*, 1992, Anderson *et al.*, 1993, Cynn *et al.*, 1995) and *ab initio* simulations regarding the cited HT-HP properties, as recently reviewed by Belmonte (2017).

MgO was here adopted as benchmark to show the capability of *QUANTAS* to employ the results coming from different *ab initio* quantum-mechanical codes and in evaluating thermodynamic and thermoelastic properties. For this reason, we performed Density Functional Theory simulations of the mineral using two well-known codes in solid-state calculations, CRYSTAL17 (Dovesi *et al.*, 2018), which employs a linear combination of atomic orbitals (LCAO) to describe the crystalline orbitals and VASP (Kresse & Furthmuller, 1996, Kresse & Hafner, 1993), which exploits projector-augmented wave (PAW) basis sets. To obtain the input data for the quasi-harmonic approximation post-processing, we calculated the total energy of the crystalline solid at 11 compression states between $0.82 \cdot V_{eq}$ and $1.12 \cdot V_{eq}$, with $V_{eq}$ the equilibrium volume. Then, a 2 × 2 × 2 supercell (with volume eight times the crystallographic cell) was modelled for each compressed/expanded state to calculate the phonon dispersion relations, for a total of 32 **k**-points of sampling. More details on the computational settings here adopted are reported in

Section S5. It is worth stressing that the present results are only for descriptive purposes. They are not converged for the supercell size, hence they may present some deviations with respect to the experimental findings of Isaak *et al.* (1989).

For the QHA post-processing of the *ab initio* data, we employed the interpolation of thermodynamic properties and the Helmholtz free energy was minimized at each pressure-temperature condition using a volume-integrated 3rd-order Birch-Murnaghan equation of state. It can be noted that thermodynamic properties, such as the isobaric heat capacity (Figure S1a) are well described, as well as the structural ones, as reported for the volumetric thermal expansion coefficient (Figure S1b). There is some absolute deviation of about 5 GPa with respect to the isothermal bulk modulus (Figure S1c), which is mainly due to the employed PBE functional (Perdew *et al.*, 1996) that is known underestimating bulk moduli of solids. However, the trend of the bulk modulus as a function of temperature is correctly described.

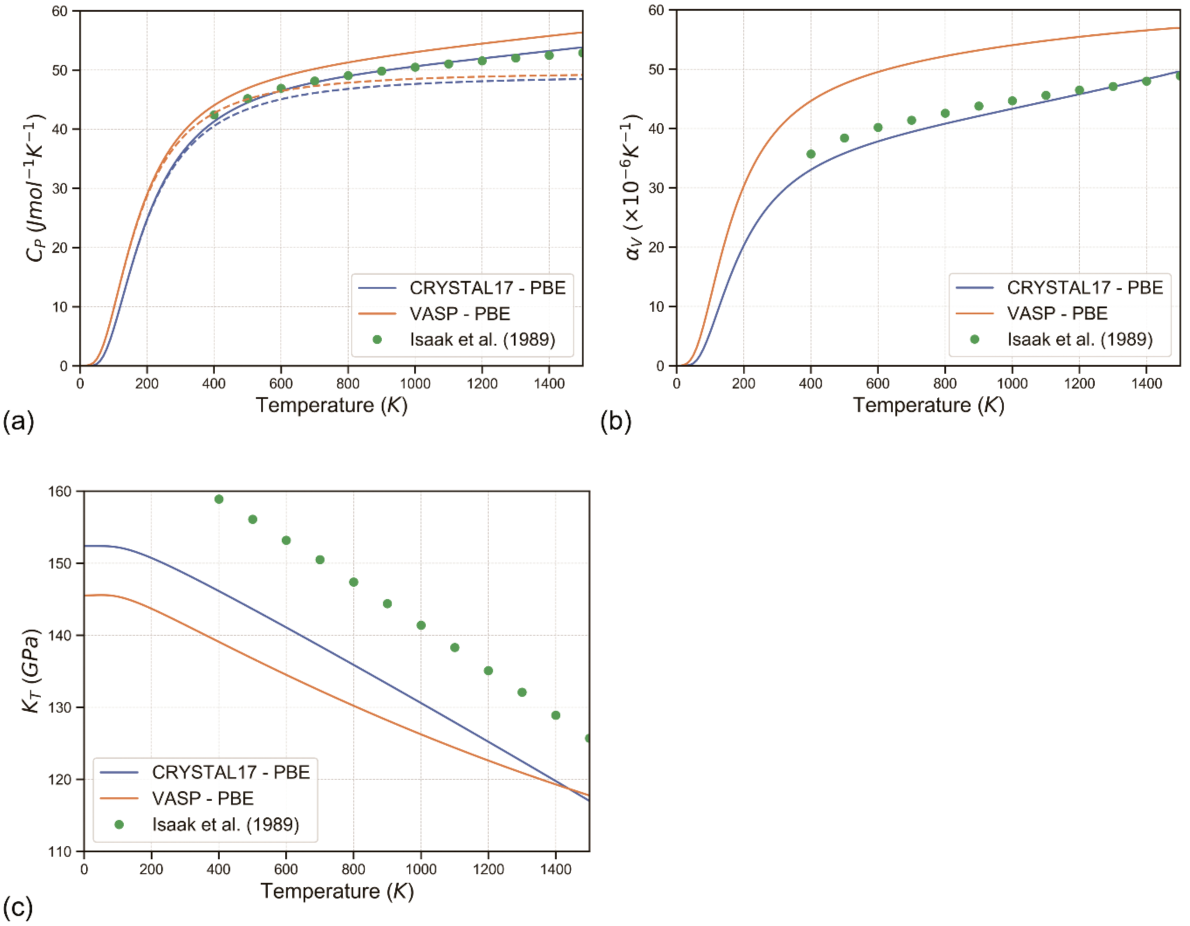


**Figure S1** (a) Isobaric ($C_P$, solid lines) and isochoric ($C_V$, dashed lines) heat capacity, (b) thermal expansion coefficient $\alpha_V$ and (c) isothermal bulk modulus $K_T$ of periclase as calculated using the CRYSTAL17 (blue line) and VASP (orange line) codes at the DFT/PBE level of theory. Green solid circles are related to the experimental data of Isaak and co-workers (Isaak *et al.*, 1989).

We also calculated the elastic moduli of periclase at absolute zero (0 K) at the same DFT level, using both CRYSTAL17 and VASP codes. The elastic constants obtained with CRYSTAL17 (and VASP) were $C_{11}$ = 308.45 (272.40) GPa, $C_{12}$ = 81.19 (87.73) GPa and $C_{44}$ = 160.81 (141.10) GPa. The elastic stiffness matrix of periclase was employed as input for *QUANTAS*, which performed the analysis of second-order elastic constants calculated with both CRYSTAL17 and VASP. The elastic properties, related to the polycrystalline behaviour of the mineral, are reported in Table S1, whereas single-crystal, directional variations of Young's modulus, linear compressibility, shear modulus, Poisson's ratio and seismic wave velocities are plotted in Figure S2.

**Table S1** Elastic properties of periclase, Young's modulus ($E$), bulk modulus ($K$), shear modulus ($\mu$) and Poisson's ratio ($\upsilon$) calculated using *QUANTAS* and the elastic moduli obtained with different theoretical methods.

| | **CRYSTAL17** | | | | **VASP** | | | |
|---|---|---|---|---|---|---|---|---|
| | *K (GPa)* | *E (GPa)* | *μ (GPa)* | *υ* | *K (GPa)* | *E (GPa)* | *μ (GPa)* | *υ* |
| Voigt | 156.9 | 327.2 | 141.9 | 0.153 | 149.3 | 286.9 | 121.6 | 0.180 |
| Reuss | 156.9 | 320.0 | 137.9 | 0.160 | 149.3 | 277.3 | 116.5 | 0.190 |
| Hill | 156.9 | 323.6 | 139.9 | 0.156 | 149.3 | 282.1 | 119.0 | 0.185 |

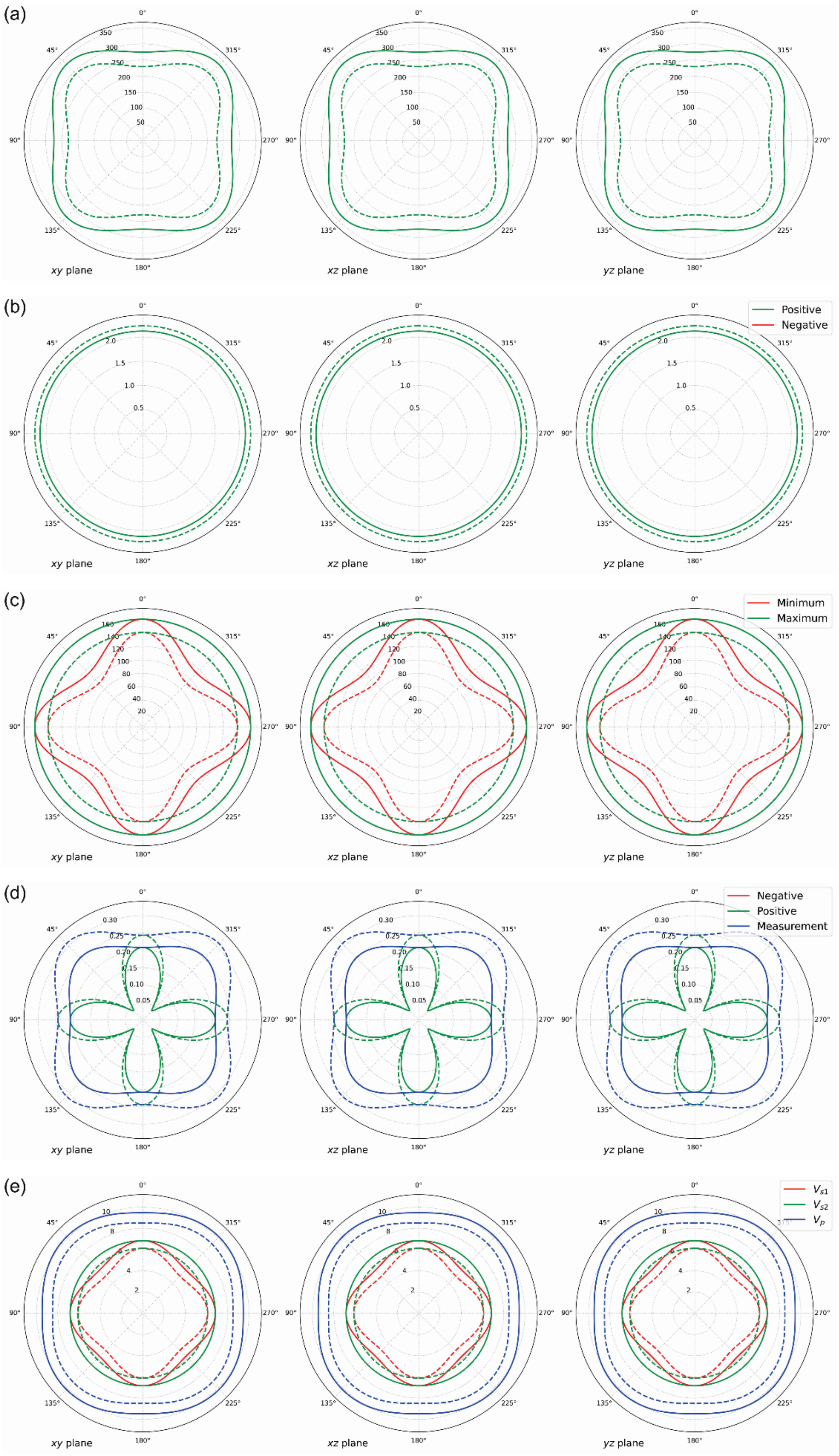


**Figure S2** Directional (*xy*, *xz* and *yz*) variations of (a) Young's modulus, (b) linear compressibility, (c) shear modulus, (d) Poisson's ratio and (e) seismic wave velocities of periclase, as calculated from *ab initio* simulations conducted with CRYSTAL17 (solid lines) and VASP (dashed lines).

## S2. Corundum α-$Al_2O_3$

Corundum (α-aluminium oxide, α-$Al_2O_3$), whose structural model is reported in Figure S3 is an extensively used material in different applications. For instance, in characterization chemistry it is used as internal reference material (SRM-720) in differential thermal analysis, because of its very interesting thermomechanical properties, such as high melting point, hardness, low solubility and density.

This mineral phase was recently simulated employing *ab initio* Density Functional Theory (DFT) methods and phonon dispersion by Erba and co-workers (2015) in order to calculate its thermodynamics and thermomechanical behaviour in a wide temperature range (0 – 2000 K) and at different pressures (0 – 12 GPa).

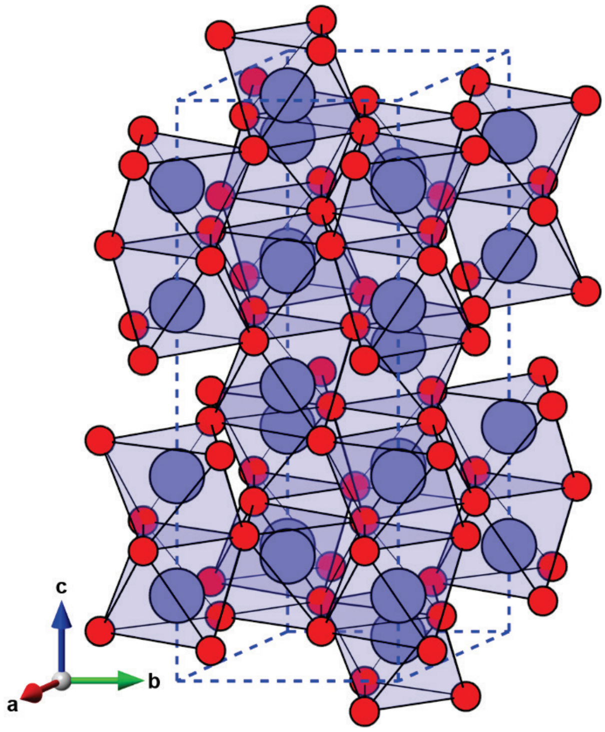


**Figure S3** Graphical model of the unit cell of α-$Al_2O_3$, space group $R\overline{3}c$. Red atoms represent oxygen, while violet ones are aluminium. $AlO_6$ octahedra are also sketched with black lines and transparent purple faces.

In the present paper, the corundum unit cell was simulated *ex novo* using the CRYSTAL17 code (Dovesi *et al.*, 2018) and the computational approach described in Section S5, which strongly adheres to the simplest one employed by Erba and co-workers (2015). In details, we considered four unit cell volumes corresponding to $1.06\cdot V_{eq}$, $1.03\cdot V_{eq}$, $1.00\cdot V_{eq}$ and $0.97\cdot V_{eq}$, with $V_{eq}$ the equilibrium volume. This means that two models are related to an expanded crystal cell, one is the equilibrium lattice and the last is a compressed state. At each volume, the phonon dispersion relations were calculated by considering 3 **k**-points in the Brillouin space, for a total of 90 vibrational frequencies. Then, the total energy and the phonon modes at each considered unit cell volume were collected in an input file for *Quantas* and

employed to calculate the thermodynamic and thermoelastic properties of the mineral phase. We considered every combination regarding the volumetric dependence of phonon properties (Section 4.2.1) and volume minimization approach (Section 4.2.2). To assess the quality of the results obtained through *QUANTAS*, the post-processed data were compared to those coming from the CRYSTAL17 code using the methods implemented via the QHA keyword (Dovesi *et al.*, 2018). It is worth remembering that the *ab initio* software uses a frequency interpolation scheme to describe the volumetric dependence of the phonon modes and the Helmholtz free energy as a function of volume is minimized using polynomial functions. The obtained quantities, while showing a trend very close to those from experiments (Anderson *et al.*, 1991), are meant only for testing purposes.

The results of the QHA calculations are reported graphically in Figure S4, showing that *QUANTAS* provides a good description of the thermal behaviour of the α-$Al_2O_3$ structure for the thermodynamics and thermoelastic quantities. A striking feature of the software is that it is perfectly in line with the quasi-harmonic results obtained with the CRYSTAL code, albeit a small difference in the behaviour of the isothermal bulk modulus can be noted for $T > 650$ K (Figure S4c). This variation could be due to the different implementation of the routines to calculate the bulk modulus.

The results reported in the inset of Figure S4a and in Figure S4c show the presence of two datasets. In fact, the volume minimization scheme, either polynomial root finding or equation of state fitting, provides some small differences in some of the calculated properties, in particular the elastic ones. This is an expected feature because polynomial root finding is a pure mathematical fitting, where the parameters do not necessarily have a physical meaning, whereas the equation of state fitting is a phenomenological approach, where each parameter is associated to a physical quantity (energy, volume, etc.). Conversely, it can be noted that there is almost no difference between the results obtained by interpolating either the phonon modes or the harmonic thermodynamic properties, as the two datasets have very small differences, as noticeable from the overlapping curves in Figure S4.

An advantage of the post-processing made by *QUANTAS* is that the proper physical treatment of the solid under investigation can be taken into account from the simulated quantum mechanical results. In fact, with most of automated procedures it is possible setting up a complex simulation in one step, which is simpler than performing several simulations, but usually at the expense of some of the available options. For example, the automated quasi-harmonic approximation analysis performed by CRYSTAL requires just the equilibrium volume of the crystalline solid and the number of compression/expansion points (Erba, 2014). Then, the routine optimizes the geometry of the solid for each hydrostatically deformed state and calculate the phonon modes at each unit cell volume. Finally, it collects the results and performs the quasi-harmonic approximation analysis. However, at present, some of the features that control the calculation of the structural and vibrational properties are missing in this automation. For example, the correction to the Hessian that takes into account the so-called longitudinal optical (LO)–transverse optical (TO) splitting of phonon modes, a phenomenon arising from the long-range Coulomb

effects due to the coherent displacement of the nuclei (Prencipe *et al.*, 2004), is not present. This is an important contribution for ionic solids, and the CRYSTAL code includes it for both Γ-point only and phonon dispersion relations outside the automated quasi-harmonic algorithm. This means that the aforementioned steps can be performed with separate simulations, one unit cell volume at a time, including all the necessary physical treatment. Then, the results can be collected and processed with external codes, such as *QUANTAS*.

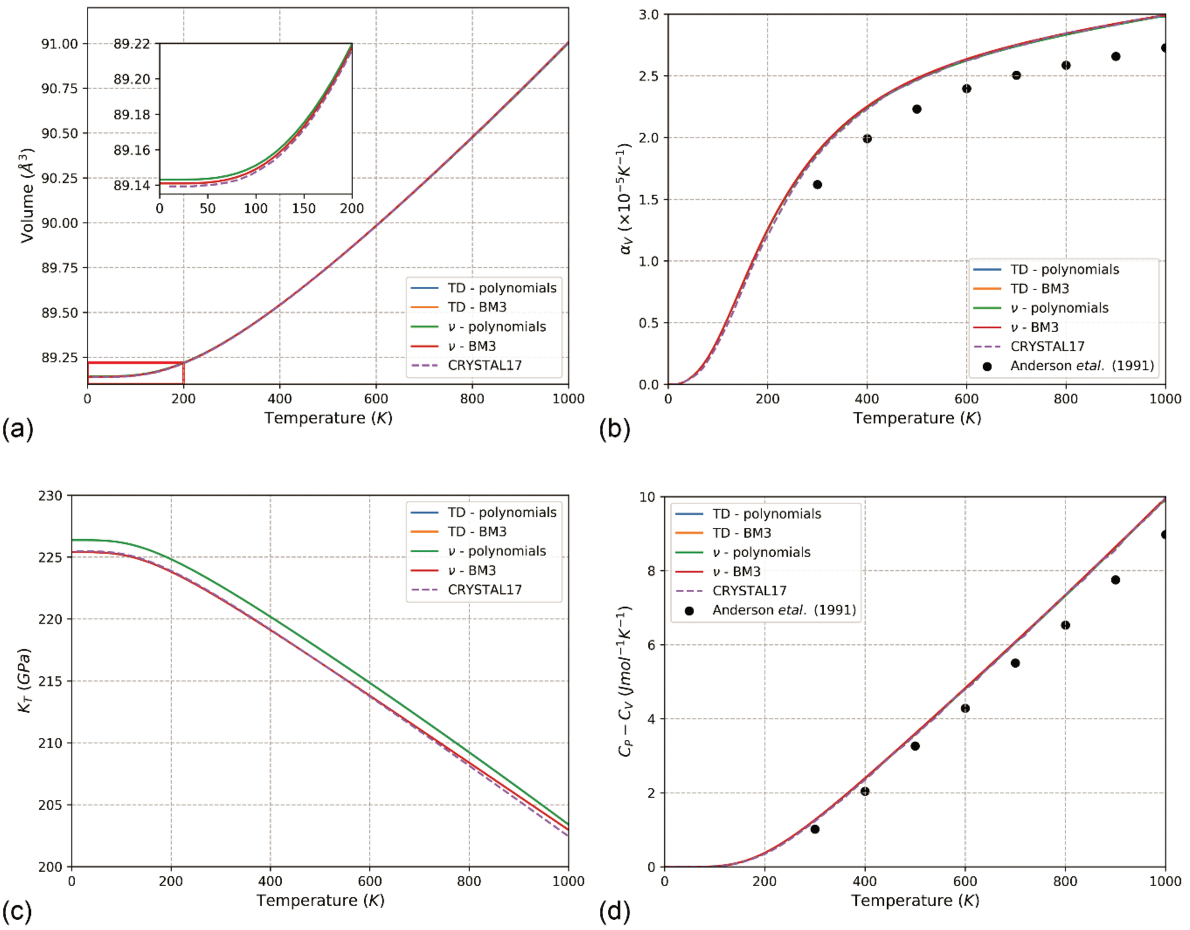


**Figure S4** Quasi-harmonic approximation results for corundum (α-$Al_2O_3$) at 0 GPa and in the temperature range 0 – 1000 K. (a) Unit cell volume, (b) coefficient of volumetric thermal expansion, (c) isothermal bulk modulus and (d) correction for isobaric heat capacity ($C_P - C_V$). Solid lines are the results obtained from *ex novo* QM simulations at the DFT/PBE level and using *QUANTAS* for post-processing of the data, whereas dashed purple lines are related to the results of Erba and co-workers (2015) calculated using CRYSTAL17 with the QHA keyword. In the legend, ν and TD indicate that the thermodynamic properties were calculated by frequency interpolation and thermodynamic interpolation, respectively, whereas the volume at *P*-*T* conditions was obtained by minimizing either polynomial function or volume-integrated 3rd-order Birch-Murnaghan equation of state (BM3). Experimental data (Anderson *et al.*, 1991) were also reported.

## S3. Phyllosilicates: pyrophyllite and muscovite 2$M_1$

Phyllosilicates are layered mineral structures presenting covalent bonds between the layers and weak interactions that hold these layers together. Phyllosilicates are interesting from the mineralogical point of view because they are water carriers in the mantle zone due to the presence of hydroxyl groups. In this perspective, pyrophyllite [$Al_2Si_4O_{10}(OH)_2$] was recently simulated at different *P-T* conditions within the quasi-harmonic approximation framework using an approach based on the mode-$\gamma$ Grüneisen's parameters (Ulian & Valdrè, 2015b). The same approach was also employed to study the 2$M_1$ polytype of muscovite [$KAl_2Si_4AlO_{10}(OH)_2$] because muscovite-paragonite series could be used as possible geothermobarometer (Ulian & Valdrè, 2015a). Very briefly, the approach employed in the cited works describes the phonon dependence on unit cell volume as $\gamma_i = -\partial \ln \nu_i / \partial \ln V$, with $\gamma_i$ the mode-$\gamma$ parameter for the *i*-th phonon mode $\nu_i$ and $V$ the unit cell volume. The mode-$\gamma$ Grüneisen's parameters were then used to calculate the thermal contribution to the unit cell pressure and then the unit cell volume at different *P-T* conditions was minimized with the *P-V* formulation of the 3rd-order Birch-Murnaghan equation of state, employing the EosFit software (Angel, 2001). Due to the large size of both pyrophyllite and 2$M_1$ polytype of muscovite, Γ-point only phonon modes were calculated for the quasi-harmonic approximation treatment. In the present work, we used the previous theoretical results of both lattice and vibrational features as input data for *QUANTAS*, to compare the quasi-harmonic approximation analysis of the code with those previously reported in the literature. Here, we used a thermodynamic interpolation scheme and a volume-integrated 3rd-order Birch-Murnaghan equation of state. The results related to pyrophyllite and muscovite are reported in Table S2 and Table S3, respectively.

The calculated 3rd-order Birch-Murnaghan parameters of pyrophyllite at 0 GPa and between 0 K and 900 K are in good agreement with those obtained by Ulian and Valdrè (2015c) with the linear mode-Grüneisen's parameters approach described by Anderson (1995). The small differences between the calculated values, particularly for the bulk modulus, can be associated to the different QHA methods previously cited. The same considerations can be extended to the muscovite case, albeit the differences between the two methods are lower than those observed for pyrophyllite (see Table S3).

**Table S2** 3rd-order Birch-Murnahan equation of state parameters of pyrophyllite, unit cell volume ($V_{0T}$), bulk modulus ($K_{0T}$) and its pressure first derivative ($K_T'$), calculated with *QUANTAS* at 0 GPa and different temperatures, compared with previous results.

| T (K) | QUANTAS | | | Previous work[1] | | |
|---|---|---|---|---|---|---|
| | $V_{0T}$ (Å$^3$) | $K_{0T}$ (GPa) | $K_T'$ | $V_{0T}$ (Å$^3$) | $K_{0T}$ (GPa) | $K_T'$ |
| 0 | 426.87 | 53.19 | 10.18 | 423.26(20) | 48.63(69) | 11.14(43) |
| 50 | 426.89 | 53.17 | 10.18 | 426.44(20) | 47.30(70) | 10.54(43) |
| 100 | 427.00 | 53.01 | 10.22 | 426.54(20) | 47.17(70) | 10.55(43) |
| 150 | 427.21 | 52.77 | 10.27 | 426.74(20) | 46.97(70) | 10.56(43) |
| 200 | 427.50 | 52.50 | 10.33 | 427.02(20) | 46.77(70) | 10.55(43) |
| 250 | 427.84 | 52.22 | 10.39 | 427.34(20) | 46.56(71) | 10.54(44) |
| 300 | 428.22 | 51.94 | 10.46 | 427.72(20) | 46.37(71) | 10.51(44) |
| 350 | 428.63 | 51.66 | 10.52 | 428.12(20) | 46.19(72) | 10.48(44) |
| 400 | 429.07 | 51.38 | 10.58 | 428.54(20) | 46.03(73) | 10.45(44) |
| 450 | 429.52 | 51.10 | 10.64 | 428.96(20) | 45.87(74) | 10.41(45) |
| 500 | 429.99 | 50.83 | 10.70 | 429.42(20) | 45.73(75) | 10.36(45) |
| 600 | 430.96 | 50.30 | 10.82 | 430.34(20) | 45.48(76) | 10.26(45) |
| 700 | 431.95 | 49.78 | 10.93 | 431.28(20) | 45.28(79) | 10.15(45) |
| 800 | 432.96 | 49.29 | 11.05 | 432.22(20) | 45.12(81) | 10.04(45) |
| 900 | 433.98 | 48.81 | 11.16 | 433.16(19) | 45.01(83) | 9.91(45) |

[1] Data from the previous DFT/B3LYP-D* analysis (Ulian & Valdrè, 2015b).

**Table S3** 3rd-order Birch-Murnaghan equation of state of the $2M_1$ polytype of muscovite, unit cell volume ($V_{0T}$), bulk modulus ($K_{0T}$) and its pressure first derivative ($K_T'$), calculated with *QUANTAS* at 0 GPa and different temperatures, compared with previous theoretical and experimental results.

| | QUANTAS | Previous work[1] | Experimental[2] |
|---|---|---|---|
| **0 K** | | | |
| $K_T$ (GPa) | 61.3 | 64.2 | – |
| $K_T'$ | 8.6 | 7.98 | – |
| $V_0$ (Å$^3$) | 929.3 | 926.86 | – |
| **573 K** | | | |
| $K_T$ (GPa) | 52.75 | 55.52 | 55.1 |
| $K_T'$ | 8.6 | 8.07 | – |
| $V_0$ (Å$^3$) | 953.5 | 950.2 | 938 |
| **723 K** | | | |
| $K_T$ (GPa) | 51.2 | 53.08 | 51.1 |
| $K_T'$ | 8.5 | 8.2 | – |
| $V_0$ (Å$^3$) | 959.2 | 956.1 | 944.1 |
| **873 K** | | | |
| $K_T$ (GPa) | 49.5 | 50.62 | 48.9 |
| $K_T'$ | 8.4 | 8.33 | – |
| $V_0$ (Å$^3$) | 965.3 | 962.4 | 952.5 |

[1] Data from (Ulian & Valdrè, 2015a)
[2] Data from (Comodi *et al.*, 2002)

### S4. Equation of state fit

For the equation of state (EoS), the performance of the orthogonal distance regression (ODR) approach used in *QUANTAS* was tested against a popular software, EosFit7 (Angel *et al.*, 2014), which is employed by both experimentalists and theoreticians to analyse their data. As a benchmark, all the formulations coded in both packages (*QUANTAS* and EOSFit7) were compared on the EoS fit of α-quartz unit cell volume at several pressure conditions measured by Angel *et al.* (1997). The fitting results obtained by including the uncertainties for both pressure and volume are reported in Table S4. There is an excellent agreement between the results provided by the two software, which means that the ODR scheme used by *QUANTAS* is as adequate in fitting the experimental data as the effective variance method employed by EosFit7. Hence, this starting benchmark on the very accurate structural data measured by Angel *et al.* (1997), with small uncertainties on both pressure and volume values, is an assessment of the stability of the proposed approach.

Then, we conducted other equation of state tests on several materials with different structures and chemistry, whose volume/lattice parameters values at different hydrostatic compressions were published in recent literature. A large collection of the results calculated with *QUANTAS* for several systems are reported in Table S5, alongside the equation of state parameters reported in previous studies (Gatta *et al.*, 2006, Gatta *et al.*, 2014, Gatta *et al.*, 2015, Kantor *et al.*, 2012, Lotti *et al.*, 2017).

To strictly compare the performance of *QUANTAS*, we adopted in our calculations the same fitting scheme (EoS formulation, order, fixed parameters, and weighting scheme) used by the various authors. It can be observed that the results are generally in very good agreement with those provided in literature. Some small differences are present, in particular when fitting lattice parameter values that exhibits a non-linear variation (*e.g.* pyrophyllite).

**Table S4** Equation of state results of *QUANTAS* and EosFit7 (Angel *et al.*, 2014) using the structural data of quartz under compression measured by Angel *et al.* (1997). The reported parameters are the bulk modulus ($K_{0T}$ ,GPa), its pressure first (*K'*, dimensionless) and second derivatives (*K''*, dimensionless), and the unit cell volume ($V_{0T}$, Å$^3$). Uncertainties on the fitting parameters are reported in parentheses. Implied (fixed) values are marked with an asterisk (*).

| **EOS formulation** | **QUANTAS** | | | | **EosFit7** | | | |
|---|---|---|---|---|---|---|---|---|
| | $K_{0T}$ | *K'* | *K''* | $V_{0T}$ | $K_{0T}$ | *K'* | *K''* | $V_{0T}$ |
| *Murnaghan* | 37.6(1) | 5.43(4) | - | 112.983(3) | 37.6(1) | 5.43(5) | - | 112.981(3) |
| *Birch-Murnaghan* | | | | | | | | |
| Order = 2 | 41.5(3) | 4* | -0.0938* | 112.97(2) | 41.5(3) | 4* | -0.0938* | 112.97(2) |
| Order = 3 | 37.1(1) | 6.00(4) | -0.2662* | 112.984(2) | 37.1(1) | 5.99(5) | -0.2648* | 112.981(2) |
| Order = 4 | 36.8(2) | 6.3(2) | -0.42(12) | 112.984(2) | 36.9(2) | 6.3(2) | -0.4(12) | 112.981(2) |
| *Natural Strain* | | | | | | | | |
| Order = 2 | 46.5(6) | 2* | -0.0215* | 112.95(5) | 46.5(6) | 2* | -0.0215* | 112.95(5) |
| Order = 3 | 36.4(1) | 6.92(7) | -0.8291* | 112.984(2) | 36.4(1) | 6.91(6) | -0.8256* | 112.981(2) |
| Order = 4 | 36.9(2) | 6.3(3) | -0.41(18) | 112.984(2) | 36.9(2) | 6.2(3) | -0.39(18) | 112.981(2) |
| *Vinet* | | | | | | | | |
| Order = 2 | 49.3(8) | 1* | -0.0068* | 112.94(6) | 49.2(8) | 1* | -0.0045* | 112.95(6) |
| Order = 3 | 37.0(1) | 6.11(4) | -0.3238* | 112.984(2) | 37.02(9) | 6.10(4) | -0.3197* | 112.981(2) |
| *Tait* | | | | | | | | |
| *Order = 2* | 41.5(3) | 4* | -0.0963* | 112.97(2) | 41.5(3) | 4* | -0.0963* | 112.97(2) |
| *Order = 3* | 37.2(1) | 5.84(4) | -0.1569* | 112.984(2) | 37.2(1) | 5.83(3) | -0.1566* | 112.981(2) |
| *Order = 4* | 36.9(2) | 6.2(2) | -0.32(10) | 112.984(2) | 36.9(2) | 6.2(2) | -0.31(11) | 112.981(2) |

**Table S5** *QUANTAS* results of different solid systems under hydrostatic compression, compared to previous EoS fit results reported in the literature from experimental data. The reported parameters are the bulk modulus ($K_{0T}$, GPa), its pressure first (*K'*, dimensionless) and second derivatives (*K''*, dimensionless), and the unit cell volume ($V_{0T}$, Å$^3$). For lattice parameters (*a*, *b*, and *c*), the axial moduli are reported as *M*, whereas their first and second pressure derivatives are *M'* and *M''*, respectively. For each system, the quantity fitted (volume, *V*, or lattice parameters, *a*, *b* and *c*), the equation of state formulation and its order are indicated in parentheses. Values marked with an asterisk (*) were kept fixed during the fitting procedure.

| System | Quantas | Reference | System | Quantas | Reference | System | Quantas | Reference |
|---|---|---|---|---|---|---|---|---|
| *Topaz (V - BM2)*[1] | | | *Pyrophyllite (V - BM2)*[2] | | | *$Cr_2O_3$ (V - BM3)*[3] | | |
| $K_{0T}$ (GPa) | 150(2) | 149(2) | $K_{0T}$ (GPa) | 56(1) | 54(1) | $K_{0T}$ (GPa) | 244(6) | 245(4) |
| *K'* | 4* | 4* | *K'* | 4* | 4* | *K'* | 3.6(2) | 3.6(2) |
| *K''* | -0.0260* | - | *K''* | -0.0727* | - | *K''* | -0.0149* | - |
| $V_{0T}$ (Å$^3$) | 345.6(1) | - | $V_{0T}$ (Å$^3$) | | 427.9(4) | $V_{0T}$ (Å$^3$) | 288.73* | 288.73* |
| *Topaz (V - BM3)*[1] | | | *Pyrophyllite (V - BM3)*[2] | | | *$Cr_2O_3$ (a - BM3)*[3] | | |
| $K_{0T}$ (GPa) | 162(3) | 158(4) | $K_{0T}$ (GPa) | 47(2) | 47(4) | $M_{0T}(a)$ (GPa) | 753(21) | 753(12) |
| *K'* | 3.0(2) | 3.3(3) | *K'* | 7.2(1.2) | 7.3(19) | *M(a)'* | 7.3(1.1) | 7.5(1) |
| *K''* | -0.0240* | - | *K''* | -0.3701* | - | *M(a)''* | -0.0243* | - |
| $V_{0T}$ (Å$^3$) | 345.51(8) | - | $V_{0T}$ (Å$^3$) | 428.3(3) | 428.3(5) | $a_{0T}$ (Å) | 4.9530* | 4.9530* |
| *Topaz (a - BM3)*[1] | | | *Pyrophyllite (a - BM2)*[2] | | | *$Cr_2O_3$ (c - BM3)*[3] | | |
| $M_{0T}(a)$ (GPa) | 433(14) | 438(15) | $M_{0T}(a)$ (GPa) | 318(18) | 297(18) | $M_{0T}$(c) (GPa) | 631(23) | 633(24) |
| *M(a)'* | 13(1) | 14(1) | *M(a)'* | 12* | 12* | M(c)' | 29(3) | 25(2) |
| *M(a)''* | -0.2131* | - | *M(a)''* | -0.2385* | - | M(c)'' | -0.0240* | - |
| $a_{0T}$ (Å) | 4.664(1) | - | $a_{0T}$ (Å) | 5.179(3) | 5.179(3) | $c_{0T}$ (Å) | 13.5884* | 13.5884* |
| *Topaz (b - BM3)*[1] | | | *Pyrophyllite (b - BM2)*[2] | | | *Colemanite (V - BM2)*[4] | | |
| $M_{0T}(b)$ (GPa) | 670(11) | 660(12) | $M_{0T}(b)$ (GPa) | 329(8) | 312(12) | $K_{0T}$ (GPa) | 70(7) | 67(4) |
| *M(b)'* | 5.5(7) | 7.8(9) | *M(b)'* | 12* | 12* | K' | 5.0(1.0) | 5.5(7) |
| *M(b)''* | -0.0111* | - | *M(b)''* | -0.2305* | - | K'' | -0.0852* | - |
| $b_{0T}$ (Å) | 8.8353(8) | - | $b_{0T}$ (Å) | 8.981(2) | 8.981(2) | $V_{0T}$ (Å$^3$) | 555(2) | 556(1) |
| *Topaz (c - BM3)*[1] | | | *Pyrophyllite (c - BM2)*[2] | | | | | |
| $M_{0T}(c)$ (GPa) | 407(10) | 396(12) | $M_{0T}(c)$ (GPa) | 80(4) | 78(3) | | | |
| *M(c)'* | 7.8(8) | 9.9(9) | *M(c)'* | 12* | 12* | | | |
| *M(c)''* | -0.0537* | - | *M(c)''* | -0.9535* | - | | | |
| $c_{0T}$ (Å) | 8.3873(11) | - | $c_{0T}$ (Å) | 9.375(14) | 9.377(13) | | | |

[1] Data from Gatta *et al.* (2006) and Gatta *et al.* (2014).
[2] Data from Gatta *et al.* (2015).
[3] Data from Kantor *et al.* (2012).
[4] Data from Lotti *et al.* (2017).
Notes: BM2 refers to a 2nd-order Birch-Murnaghan EoS, BM3 to a 3rd-order Birch-Murnaghan EoS.

### S5. Simulation methods

First principle simulations of periclase MgO were conducted using both the CRYSTAL17 (Dovesi *et al.*, 2018) and VASP codes (Kresse & Furthmuller, 1996, Kresse & Hafner, 1993), while for corundum α-$Al_2O_3$ only the former software was employed. The generalized gradient approximation PBE functional (Perdew *et al.*, 1996) was employed for all the simulations.

In CRYSTAL, multielectron wave functions are constructed as an antisymmetrized product (Slater determinant) of monoelectronic crystalline orbitals (CO) that are linear combination of local functions (atomic orbitals, AO) centred on each atom in the system. In turn, atomic orbitals (basis set) are linear combinations of Gaussian-type functions (GTF). We employed the all-electron basis sets consisting of atom-centred GTF of triple-ζ valence plus polarization quality (Peintinger *et al.*, 2013). The exchange–correlation contribution is performed over a pruned grid given by 75 points and 974 angular points (XLGRID) and obtained from the Gauss–Legendre quadrature and Lebedev schemes (Prencipe *et al.*, 2004). This is a good compromise between accuracy and cost of calculation for geometry optimization and vibrational frequencies. The tolerance thresholds that control accuracy of the Coulomb and exchange integrals were set to $10^{-6}$ and $10^{-14}$, respectively (Dovesi *et al.*, 2018). The Hamiltonian matrix has been diagonalized using a shrinking factor that leads to 72 and 13 reciprocal lattice points (*k*-points) for periclase and corundum, respectively. The convergence of total energy was reached when the difference between the energy of two subsequent self-consistent field cycles was less than $10^{-8}$ Hartree for all geometry optimizations and to $10^{-10}$ Hartree for phonon frequency calculations. For the calculation of the quasi-harmonic properties, the lattice parameters and atomic positions were optimized in the same run and, after the relaxation procedure, the vibrational frequencies were calculated using the phonon dispersion approach (Pascale *et al.*, 2004, Tosoni *et al.*, 2005). Second-order elastic constants (SOECs) were calculated using the automated procedure described by Perger and co-workers (Perger, 2010, Perger *et al.*, 2009).

VASP employs plane waves basis sets, and we chose the projector augmented-wave approach described by Kresse and Joubert (Kresse & Joubert, 1999). In this case, a single parameter, *i.e.* the kinetic energy cutoff controls the quality of the basis sets, and we employed a value of 600 eV that is more than adequate for both structural optimizations and phonon frequency calculations. The geometry optimizations were performed using the conjugated gradient algorithm, with a tolerance of $10^{-4}$ eV/Å. A Monkhorst-Pack grid of 72 *k*-points was employed to sample the first Brillouin zone (Monkhorst & Pack, 1976). Phonon dispersion relations in VASP were calculated using the phonopy code (Togo & Tanaka, 2015).

## References


Anderson, O. L., Isaak, D. L. & Oda, H. (1991). *J Geophys Res-Sol Ea* **96**, 18037-18046.
Anderson, O. L., Oda, H., Chopelas, A. & Isaak, D. G. (1993). *Physics and Chemistry of Minerals* **19**, 369-380.
Anderson, O. L., Oda, H. & Isaak, D. (1992). *Geophysical Research Letters* **19**, 1987-1990.
Angel, R. J., Allan, D. R., Milletich, R. & Finger, L. W. (1997). *Journal of Applied Crystallography* **30**, 461-466.
Angel, R. J., Gonzalez-Platas, J. & Alvaro, M. (2014). *Zeitschrift Fur Kristallographie* **229**, 405-419.
Belmonte, D. (2017). *Minerals* **7**.
Chopelas, A. (1990). *Physics and Chemistry of Minerals* **17**, 142-148.
Comodi, P., Gatta, G. D., Zanazzi, P. F., Levy, D. & Crichton, W. (2002). *Physics and Chemistry of Minerals* **29**, 538-544.
Cynn, H., Anderson, O. L., Isaak, D. G. & Nicol, M. (1995). *Journal of Physical Chemistry* **99**, 7813-7818.
Dovesi, R., Erba, A., Orlando, R., Zicovich-Wilson, C. M., Civalleri, B., Maschio, L., Rerat, M., Casassa, S., Baima, J., Salustro, S. & Kirtman, B. (2018). *Wires Comput Mol Sci* **8**, E1360.
Erba, A. (2014). *Journal of Chemical Physics* **141**, 124115.
Gatta, G. D., Lotti, P., Merlini, M., Liermann, H.-P., Lausi, A., Valdrè, G. & Pavese, A. (2015). *Physics and Chemistry of Minerals* **42**, 309-318.
Gatta, G. D., Morgenroth, W., Dera, P., Petitgirard, S. & Liermann, H. P. (2014). *Physics and Chemistry of Minerals* **41**, 569-577.
Gatta, G. D., Nestola, F. & Ballaran, T. B. (2006). *Physics and Chemistry of Minerals* **33**, 235-242.
Isaak, D. G., Anderson, O. L. & Goto, T. (1989). *Physics and Chemistry of Minerals* **16**, 704-713.
Kantor, A., Kantor, I., Merlini, M., Glazyrin, K., Prescher, C., Hanfland, M. & Dubrovinsky, L. (2012). *American Mineralogist* **97**, 1764-1770.
Kresse, G. & Furthmuller, J. (1996). *Computational Materials Science* **6**, 15-50.
Kresse, G. & Hafner, J. (1993). *Physical Review B* **48**, 13115-13118.
Kresse, G. & Joubert, D. (1999). *Physical Review B* **59**, 1758-1775.
Lotti, P., Gatta, G. D., Comboni, D., Guastella, G., Merlini, M., Guastoni, A. & Liermann, H. P. (2017). *Journal of the American Ceramic Society* **100**, 2209-2220.
Monkhorst, H. J. & Pack, J. D. (1976). *Physical Review B* **8**, 5188-5192.
Pascale, F., Zicovich-Wilson, C. M., Gejo, F. L., Civalleri, B., Orlando, R. & Dovesi, R. (2004). *Journal of Computational Chemistry* **25**, 888-897.
Peintinger, M. F., Oliveira, D. V. & Bredow, T. (2013). *Journal of Computational Chemistry* **34**, 451-459.
Perdew, J. P., Burke, K. & Ernzerhof, M. (1996). *Physical Review Letters* **77**, 3865-3868.
Perger, W. F. (2010). *International Journal of Quantum Chemistry* **110**, 1916-1922.
Perger, W. F., Criswell, J., Civalleri, B. & Dovesi, R. (2009). *Comput Phys Commun* **180**, 1753-1759.
Prencipe, M., Pascale, F., Zicovich-Wilson, C. M., Saunders, V. R., Orlando, R. & Dovesi, R. (2004). *Physics and Chemistry of Minerals* **31**, 559-564.
Togo, A. & Tanaka, I. (2015). *Scripta Mater* **108**, 1-5.
Tosoni, S., Pascale, F., Ugliengo, P., Orlando, R., Saunders, V. R. & Dovesi, R. (2005). *Mol. Phys.* **103**, 2549-2558.
Ulian, G. & Valdrè, G. (2015a). *American Mineralogist* **100**, 935-944.
Ulian, G. & Valdrè, G. (2015b). *Physics and Chemistry of Minerals* **42**, 609-627.
